# Risk categorization for calculating net reclassification improvement

**DOI:** 10.1007/s10654-013-9824-9

**Published:** 2013-07-10

**Authors:** Mitsuyoshi Takahara, Naoto Katakami, Hideaki Kaneto, Iichiro Shimomura

**Affiliations:** Department of Metabolic Medicine, Osaka University Graduate School of Medicine, 2-2 Yamadaoka, Suita, 565-0871 Osaka Japan

We read with great interest a recent report by Mühlenbruch and colleagues [1], clearly demonstrating the dependency of the net reclassification improvement (NRI) on risk categories. Their results underlined the recommendation to calculate NRI based on a priori meaningful risk categories that are linked to clinical decision-making [2, 3]. They also reconfirmed another original recommendation to use three categories, i.e., low, intermediate, and high risk [2]. However, many medical fields so far lack such firmly established three-class categories, and leave room for an arbitrary and intentional categorization for calculating NRI in clinical studies of risk assessment. Here we address this issue by proposing an alternative to the original NRI, which uses a valid three-class categorization based on a familiar concept, the likelihood ratio (LR).

Likelihood ratio is a familiar statistical methodology for assessing the performance of a diagnostic test [4, 5]. LR equals the fold difference of the post-test odds from the pre-test odds, showing whether a test will provide any meaningful change in the probability that a disease exists. LR > 1 indicates increased post-test probability of the disease, and therefore a positive finding rules in a diagnosis. On the other hand, LR < 1 indicates decreased post-test probability, and thus a negative finding rules out a diagnosis. Generally, LR for a positive finding (LR+) of at least 2 and that for a negative finding (LR−) of at most 0.5 (i.e., 2^−1^) are mentioned as meaningful changes in probability [4, 5].

Here we apply this concept of LR to the risk assessment. All we intend to do is to see whether the post-test probability of a disease is meaningfully increased from the pre-test probability in the diseased subjects and is meaningfully decreased in the healthy subjects. This is the very concept of LR. When pre-test odds equal *Q*_0_ and a priori desired LR+ and LR− are >*D* and <*D* ^−1^, the desired post-test odds are calculated as >*Q*_0_ × *D* for the positive finding and as <*Q*_0_ × *D* ^−1^ for the negative finding. Since the odds correspond one-to-one with the probabilities, the desired post-test probability can be determined by *D* and the pre-test probability *P*_0_.

In a risk score with a higher predictive performance, a larger number of subjects are expected to have the desired post-test probability (i.e., the diseased subjects have sufficiently high post-test probabilities, and the healthy subjects have sufficiently low post-test probabilities). We can therefore compare two risk scores by assessing which risk score assigns more subjects with the desired post-test probability. This is the very concept of the NRI using the desired post-test probability for risk categorization.

To illustrate the practical use of this LR-based NRI, we examined as an example whether adding hemoglobin A1c (HbA1c) to fasting plasma glucose (FPG), age, and body mass index would improve the screening performance of diabetes mellitus (DM) in men, using a database of 1,404 male Japanese employees (UMIN000002391). All the subjects had FPG levels <7.0 mmol/l and underwent a 75-g oral glucose tolerance test (OGTT), revealing that 79 subjects (6 %) had DM (i.e., 120-min plasma glucose levels ≥11.1 mmol/l). Note that the current example was intended to illustrate the proposed concept, rather than serve as a substantive analysis in search of a prediction model. We first estimated the probability of DM in each subject by the following two logistic regression models: a model in which FPG, age, and BMI were entered (FPG + Age + BMI model), and a model in which HbA1c was additionally entered (FPG + Age + BMI + HbA1c model). In the logistic regression analysis, HbA1c was associated with the presence of DM, independently of FPG, age and BMI (*p* < 0.001); its adjusted odds ratio was 2.6 (95 %CI 2.0–3.5) per one SD (i.e., 0.5 %) increase. We thereafter investigated whether the FPG + Age + BMI + HbA1c model had a higher predictive performance than the FPG + Age + BMI model, using the LR-based NRI. The pre-test probability *P*_0_ was 6 %, and the standards of LR + and LR− were set as 2 and 0.5 (i.e., *D* = 2). The desired post-test probability was then calculated as >11 % for the positive finding and <3 % for the negative finding. We therefore defined the risk categories of the post-test probability as follows: “low risk” (<3 %), “intermediate risk” (3–11 %), and “high risk” (>11 %). Table 1 is the reclassification table based on the LR-based categorization. The NRI of the FPG + Age + BMI + HbA1c model from the FPG + Age + BMI model was +0.161 (*p* = 0.004) for the overall population, +0.063 (*p* = 0.251) for the diabetic subjects, and +0.098 (*p* < 0.001) for the non-diabetic subjects [2]. Note that the increment in the *C* statistic was +0.034 (*p* = 0.005), from 0.883 in the FPG + Age + BMI model and 0.917 in the FPG + Age + BMI + HbA1c model.

**Table 1:** Reclassification by the addition of HbA1c in the risk assessment for OGTT-detected DM in men

	FPG + Age + BMI + HbA1c model		
	Low risk (<3 %)	Intermediate risk (3–11 %)	High risk (>11 %)
Subjects with OGTT-detected DM (*n* = 79)			
FPG + Age + BMI model			
Low risk (<3 %)	3 (4 %)	5 (6 %)	1 (1 %)
Intermediate risk (3–11 %)	2 (3 %)	5 (6 %)	6 (8 %)
High risk (>11 %)	0 (0 %)	5 (6 %)	52 (66 %)
Subjects without OGTT-detected DM (*n* = 1,325)			
FPG + Age + BMI model			
Low risk (<3 %)	857 (65 %)	37 (3 %)	1 (0 %)
Intermediate risk (3–11 %)	129 (10 %)	131 (10 %)	18 (1 %)
High risk (>11 %)	3 (0 %)	54 (4 %)	95 (7 %)

Data are number (percentage) of subjects. High risk (>11 %) was equivalent to the post-test probability providing LR larger than 2, whereas low risk (<3 %) was that providing LR smaller than 0.5 (i.e., 2^−1^). The NRI of the FPG + Age + BMI + HbA1c model from the FPG + Age + BMI model was +0.161 (*p* = 0.004) for the overall population, +0.063 (*p* = 0.251) for subjects with OGTT-detected DM, and +0.098 (*p* < 0.001) for those without it [2]. To demonstrate the current example, we used a database of male Japanese employees in the Amagasaki Visceral Fat Study (UMIN000002391). Approval of the human ethics committee of Osaka University, and written informed consent from every participant were obtained

In this letter, we propose the LR-based NRI, as an alternative to the original NRI [2]. The LR-based NRI preserves the use of categories, or, in other words, its calculation is based on three-category “classification,” without losing the original concept of “reclassification.” This is in contrast to the continuous (i.e., category-free) NRI, another and a well-established alternative to the original NRI. In the current example, the pre-test probability *P*_0_ was derived from the prevalence of the outcome in the study sample. However, it may be valid to use a known value of the target population in some demands. In addition, we used LR+ of 2 and LR− of 0.5 in the example, but in some scenarios, other standards (e.g., 5 or 10 of LR+, and 0.2 or 0.1 of LR−) might be used if there is a good reason. When researchers demonstrate the LR-based NRI, the clear statement of which standards of LR they use will be required. The statement will help the readers correctly interpret their findings, on the basis of the established understanding of LR. LR is a familiar concept and would be a common language between the researchers and the readers, in discussion of whether the LR standards are valid. This is considered an advantage of using the concept of LR.

In conclusion, we proposed an NRI based on the concept of LR, with potential use in various fields requiring risk assessment.
